# Shiga Toxin 2a Binds to Complement Components C3b and C5 and Upregulates Their Gene Expression in Human Cell Lines

**DOI:** 10.3390/toxins13010008

**Published:** 2020-12-24

**Authors:** Sára Kellnerová, Sneha Chatterjee, Rafael Bayarri-Olmos, Louise Justesen, Heribert Talasz, Wilfried Posch, Samyr Kenno, Peter Garred, Dorothea Orth-Höller, Marco Grasse, Reinhard Würzner

**Affiliations:** 1 Institute of Hygiene and Medical Microbiology, Medical University of Innsbruck, Schöpfstraβe 41, A-6020 Innsbruck, Austria; sara.kellnerova@i-med.ac.at (S.K.); sneha.chatterjee@alumni.i-med.ac.at (S.C.); wilfried.posch@i-med.ac.at (W.P.); samyr.kenno@alumni.i-med.ac.at (S.K.); marco.grasse@i-med.ac.at (M.G.); 2 Laboratory of Molecular Medicine, Department of Clinical Immunology Section 7631, Rigshospitalet, University of Copenhagen, Ole Maaloesvej 26, 2200 Copenhagen, Denmark; rafael.bayarri.olmos@regionh.dk (R.B.-O.); louise.justesen.01@regionh.dk (L.J.); peter.garred@regionh.dk (P.G.); 3 Centre of Chemistry and Biomedicine, Division of Clinical Biochemistry, Medical University of Innsbruck, Innrain 80, A-6020 Innsbruck, Austria; Heribert.Talasz@i-med.ac.at

**Keywords:** Shiga toxin 2a, hemolytic uremic syndrome, C3, C5, intracellular complement, HK-2, HCT-8

## Abstract

Enterohemorrhagic *Escherichia coli* (EHEC) infections can cause EHEC-associated hemolytic uremic syndrome (eHUS) via its main virulent factor, Shiga toxins (Stxs). Complement has been reported to be involved in the progression of eHUS. The aim of this study was to investigate the interactions of the most effective subtype of the toxin, Stx2a, with pivotal complement proteins C3b and C5. The study further examined the effect of Stx2a stimulation on the transcription and synthesis of these complement proteins in human target cell lines. Binding of Stx2a to C3b and C5 was evaluated by ELISA. Kidney and gut cell lines (HK-2 and HCT-8) were stimulated with varied concentrations of Stx2a. Subsequent evaluation of complement gene transcription was studied by real-time PCR (qPCR), and ELISAs and Western blots were performed to examine protein synthesis of C3 and C5 in supernatants and lysates of stimulated HK-2 cells. Stx2a showed a specific binding to C3b and C5. Gene transcription of C3 and C5 was upregulated with increasing concentrations of Stx2a in both cell lines, but protein synthesis was not. This study demonstrates the binding of Stx2a to complement proteins C3b and C5, which could potentially be involved in regulating complement during eHUS infection, supporting further investigations into elucidating the role of complement in eHUS pathogenesis.

## 1. Introduction

Enterohemorrhagic *Escherichia coli* (EHEC) is the most common cause of a life-threatening complication known as hemolytic uremic syndrome (HUS). With long-term sequelae [1,2], it is typically characterized by microangiopathic anemia, thrombocytopenia and acute renal failure [3].

Shiga toxins (Stxs) are the predominant virulence factors involved in EHEC-associated hemolytic uremic syndrome (eHUS) [4,5]. Upon oral ingestion, the EHEC bacteria colonize the gut, releasing Stx, which is then translocated into the circulation, ultimately reaching its target organs, mainly the kidneys [5,6]. Stx binds to the globotriaosylceramide, GB3 or GB4 receptors present in the kidneys [7], triggering the internalization of the toxin, which generally leads to inhibition of protein synthesis within target cells [8]. Stxs are categorized into two types of immunologically distinct toxins—Stx1 and Stx2. Stx2, and particularly the Stx2a subtype, is most commonly associated with severe disease [9,10].

In addition to Shiga-toxin-associated eHUS or typical HUS, a hereditary form of HUS —called atypical HUS (aHUS)—has also been characterized. aHUS is associated with the dysregulation of complement genes, leading to the uncontrolled activation of the complement system [11]. Complement overreaction was also observed in the acute phase of typical HUS [12]. This overreaction is considered to aggravate the pathophysiological consequences of eHUS by, among other aspects, promoting endothelial prothrombotic state as well as contributing to podocyte dysfunction and loss. Complement involvement in eHUS pathogenesis was also reported [13,14] and further supported by the results of clinical studies that have demonstrated positive effects with the usage of the C5 complement inhibitor eculizumab, an anti-C5 antibody, in aHUS and eHUS patients [15,16]. Interestingly, Stx2a has been shown to have the ability to activate complement in serum of healthy donors and to influence some complement regulatory components, altering complement function [13,17]. Nevertheless, there remains a great gap in our understanding of the role of Stx2a and complement in eHUS pathogenesis.

The complement system is a proteolytic cascade composed of soluble and membrane-bound proteins that are present in plasma, tissues and within cells [18]. Its three distinct pathways—classical (CP), lectin (LP), and alternative (AP)— result in the cleavage/activation of the unstable central complement component, C3, into C3b and C3a. C3b is involved in opsonizing surface structures or in the cleavage and subsequent activation of another central, but stable, complement component, C5. C5 cleavage results in the generation of C5a and C5b. The latter is then involved in the assembly of the C5b-9/terminal complement complex (TCC), or membrane attack complex (MAC). The formation of MAC on cell surfaces triggers cell lysis and the consequent induction of inflammatory responses. There are several co-factors that are known to promote the complement activation, such as factor B (fB) or factor P (fP), or inhibit complement activation, such as factor H (fH) or factor I (fI) [19,20].

Although complement proteins are mainly produced by the liver, it has been demonstrated that complement components such as C3, C4 and fB can also be locally synthesized in the human intestine in vitro [21,22]. Additionally, some studies have shown extrahepatic synthesis by various cells including monocytes/macrophages, lymphocytes [23], fibroblasts, endothelial [24,25] and epithelial cells [21,26,27], thereby contributing to complement regulation. Consequently, complement production and/or regulation in extrahepatic target organs during eHUS infection should be examined.

The aim of this study was to investigate the interaction of Stx2a with C3b and C5 and to further investigate whether Stx2a has an ability to regulate complement proteins in extrahepatic target cell lines. For this purpose, human proximal tubular epithelial kidney cell line HK-2 and the human colon epithelial HCT-8 cell line, both susceptible and targets of Stx2a, were used. We demonstrated the binding of Stx2a to C3b and C5. Moreover, our results showed upregulation of the transcription of these two complement proteins in susceptible extrahepatic cell lines upon stimulation with Stx2a. However, this mRNA upregulation was not translated on a protein level, most likely due to protein synthesis inhibition by the Stx2a. Ultimately, these findings may further elucidate the interaction between complement and Stx2a in the pathogenesis of eHUS.

## 2. Results

### 2.1. Stx2a Binds to Complement C3b and C5

**Figure 1:**
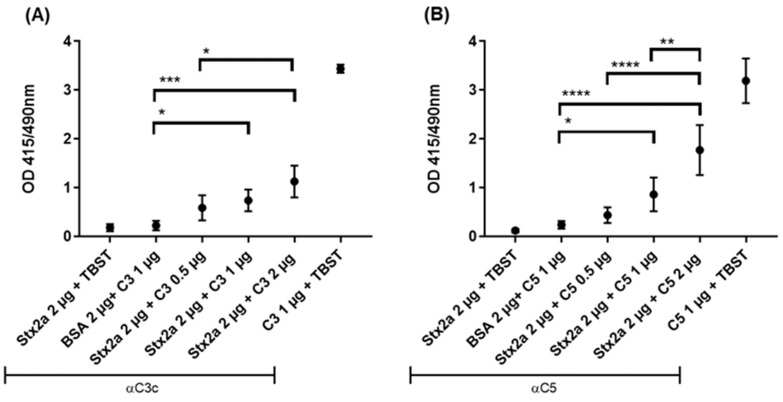
Stx2a binding to C3b and C5. Optical density (OD) values of indirect ELISA assessing the specific binding between Stx2a and C3b (**A**) or C5 (**B**). Two negative controls were used, in one Stx2a was replaced with bovine serum albumin (BSA) as a coating agent, while in the other, tween buffer (TBST) was added instead of complement proteins. Rabbit anti-human C3c antibody (Ab) was used to detect C3b and rabbit anti-human C5 Ab to detect C5. Directly coated C3b or C5 were used as positive controls. Means ± SDs are shown (*n* = 5). One-way ANOVA was applied for the inter-comparison of Stx2a groups in which C3b or C5 were added and the BSA-coated group, following Tukey correction with * *p* < 0.05, ** *p* < 0.01, *** *p* < 0.001 and **** *p* < 0.0001.

Binding of Stx2a to complement C3b or C5 was assessed by ELISA. Stx2a was coated onto the solid phase of microtiter plates followed by incubation with C3b (as uncleaved C3 is not stable enough) or C5 diluted in tween buffer (TBST). When equal or greater amounts than 1 µg of C3b or C5 were added, both C3b (Figure 1A) and C5 (Figure 1B) showed a significant, specific binding to Stx2a upon detection with anti-human C3c and anti-human C5 antibodies, respectively, when compared to the negative control—bovine serum albumin (BSA) coated wells, incubated with C3b/C5 and detected with anti-C3c/C5 antibodies.

### 2.2. Stx2a Upregulates C3 and C5 mRNA Expression in HK-2 and HCT-8 Cells

**Figure 2:**
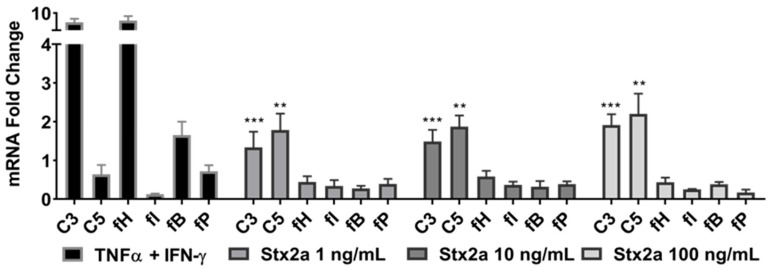
C3 and C5 mRNA upregulation in HK-2 cell line. Evaluated qPCR analyses of mRNA expression of specific complement genes in HK-2 cells after stimulation for 6 h with increasing concentrations of Stx2a. qPCR data were normalized against *TOP2B* and *GAPDH*, and the fold change ± SD is shown (*n* = 3). Unpaired Student’s *t* test was applied to compare untreated cells with the treatment groups, following Bonferroni–Holm correction with ** *p* < 0.01 and *** *p* < 0.001.

Complement production was studied in the proximal tubular epithelial kidney cell line HK-2 that was stimulated with 1, 10 or 100 ng/mL of Stx2a for 6 h. mRNA expression studies by real-time PCR (qPCR) revealed a significant increase in C3 and C5 mRNA expression at Stx2a concentrations of 1, 10 and 100 ng/mL, in comparison with untreated cells (Figure 2). The transcriptomes of the complement proteins fH, fI, fB and fP were not affected by Stx2a stimulation. Stimulation with TNF-α and IFN-γ—positive control for triggering C3, fB and fH production in human proximal tubular cells [28,29]—highly induced C3 and fH mRNA expression, and to a lesser extent, the mRNA expression of C5, fB and fP.

**Figure 3:**
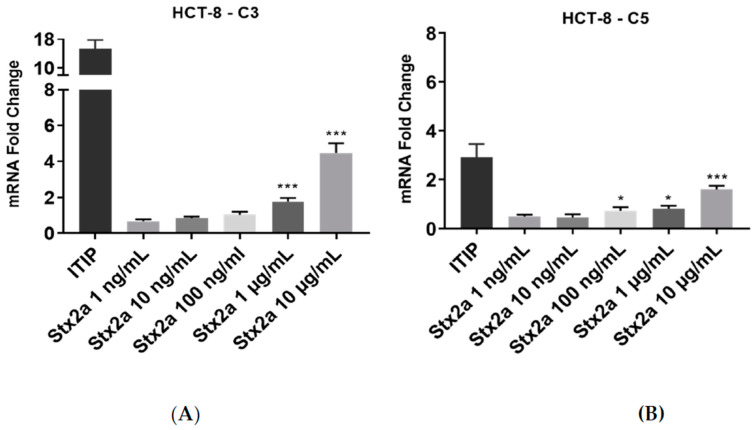
C3 and C5 mRNA expression in HCT-8 cell line. Evaluated qPCR analyses of C3 (**A**) and C5 (**B**) mRNA expression in HCT-8 cells after stimulation for 6 h with increasing concentrations of Stx2a. Data were normalized against *GAPDH*, and the fold change of a sample size of *n* = 4 ± SD is shown. Unpaired Student’s *t* test was applied to compare untreated cells with the with the treatment groups, following Bonferroni–Holm correction with * *p* < 0.05 and *** *p* < 0.001.

Increasing Stx2a concentrations were also applied to human colon epithelial HCT-8 cells for 6 h. There was no significant change in expression of C3 mRNA at Stx2a concentrations ranging from 1 to 100 ng/mL. The same was true for the expression of C5 transcript at Stx2a concentrations ranging from 1 to 10 ng/mL. HCT-8 cells showed a slight upregulation in C5 mRNA expression upon stimulation with 100 ng/mL of Stx2a. To observe whether HCT-8 cells require stimulation with higher Stx2a concentrations to upregulate complement transcripts, the cells were treated with 1 and 10 µg/mL Stx2a for 6 h. This led to a significantly higher mRNA expression of C3 and C5, where stimulation with 10 µg/mL yielded the greatest increases in mRNA expression (Figure 3). Cells were treated with a standardized cocktail of pro-inflammatory cytokines used for monocytes maturation—ITIP (10^3^ U/mL IL-1β, 10 ng/mL TNF-α, 10^3^ U/mL IL-6 and 1 µg/mL PGE-2) [22,30]—which contained three cytokines previously described as inducers of complement components expression in human intestinal epithelial cells [31]. The treatment with ITIP resulted in an upregulation of complement C3 and C5 mRNA, being stronger for C3.

### 2.3. Complement Protein C3 and C5 Synthesis in HK-2 Cells

As HK-2 cells are physiologically nearest to the target cells and also appeared to be more sensitive to Stx2a-induced mRNA upregulation, they were used to study the complement protein expression. For that, they were either stimulated with different Stx2a concentrations from 0.001 to 10 µg/mL, with a combination of TNF-α and IFN-γ as positive control for C3 production [28], or they remained unstimulated for 9 h. C3 and C5 production in the cell lysates or supernatants was analyzed by ELISA and Western blot. As additional positive controls, unstimulated samples were spiked with 325 ng of pure C3b or C5, while pure medium (for supernatant) or lysis buffer (for cell lysates) were used as negative controls. Furthermore, the cut-off value for the ELISA assays was established in reference to the negative control [32]. The spiked samples and negative controls were excluded from the statistical analysis.

**Figure 4:**
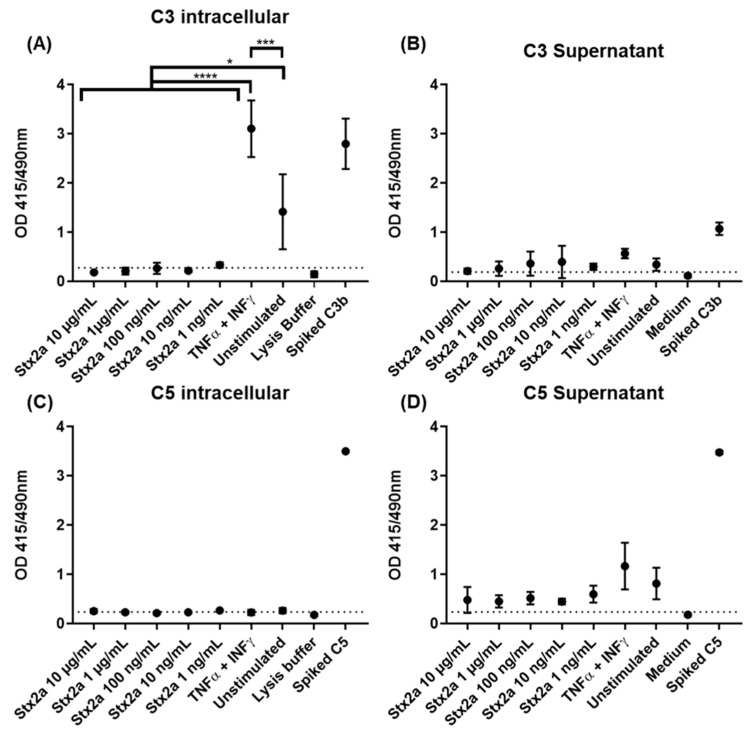
C3 and C5 protein synthesis in HK-2 cell line. OD values of sandwich ELISA assessing intracellular (**A**,**C**) and extracellular (**B**,**D**) protein expression of C3 (**A**,**B**) and C5 (**C**,**D**) in HK-2 cells after 9 h stimulation with increasing concentrations of Stx2a (0.001–10 µg/mL) are shown. Several controls have been performed: unstimulated cells (unstimulated), TNF-α- and IFN-γ-stimulated cells as positive control, lysis buffer or medium as negative controls and lysate or concentrated supernatant of unstimulated cells spiked with 325 ng of C3b and C5 (Spiked C3b/C5) as additional positive controls. Dashed line indicates the cut-off of the ELISA (mean of the negative control + 2.6 times its SD). Means ± SDs with a sample size of *n* = 3 are shown. One-way ANOVA was applied for the comparison of all groups excluding spiked and negative controls, following Tukey correction with * *p* < 0.05, *** *p* < 0.001 and **** *p* < 0.0001.

Complement protein C3 was found by ELISA in recovered cell lysates from TNF-α and IFN-γ stimulated and unstimulated cells, but only in a few lysates from cells stimulated with Stx2a. TNF-α- and IFN-γ-stimulated cells showed significantly higher C3 protein levels compared to unstimulated cells, indicating that C3 production can be achieved and induced in HK-2 cells. However, cells stimulated with Stx2a (0.001–10 µg/mL) did not produce C3 in amounts above the detection limit of our current methods. This means that the cells produced either no, or at the very least, significantly lower amounts of C3 than both the TNF-α and IFN-γ stimulated and the unstimulated cells (Figure 4A). This may point towards a potential ability of Stx2a to inhibit C3 protein synthesis in these cells. Complement protein C3 was also found in almost all recovered and concentrated supernatants, following the same protein profile. However, in this case, no significant differences for C3 presence were observed for any of the groups (Figure 4B). Although complement protein C5 was not detected by ELISA in the cell lysates of most of the Stx2a-stimulated cells, TNF-α- and IFN-γ-stimulated cells and unstimulated cells (Figure 4C), it was found in all recovered and concentrated supernatants. C5 followed a similar protein profile to C3, however, none of the groups showed significant differences in C5 protein levels (Figure 4D). Given that we have not observed any upregulation for C5 production, we can confirm that none of the applied stimuli can regulate its production in HK-2 cells.

**Figure 5:**
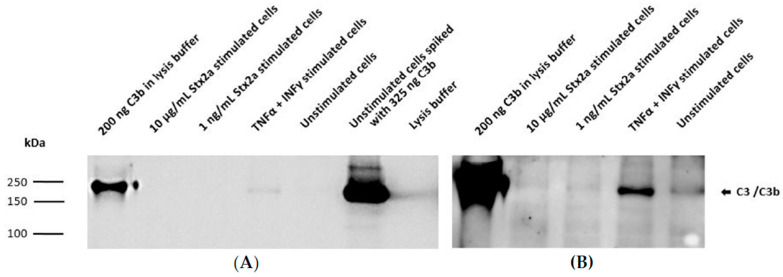
Intracellular protein synthesis of C3 by HK-2 cell line. Western blot results assessing intracellular protein expression of C3 in HK-2 cells after stimulation for 9 h with 1 ng/mL or 10 µg/mL Stx2a. The presence of C3 was also studied in concentrated lysates of TNF-α- and IFN-γ-stimulated cells and unstimulated cells. Unstimulated cells spiked with 325 ng C3b or 200 ng of pure C3b were used as positive controls, while lysis buffer was used as a negative control. Mouse anti-human C3 antibody (Ab) was used to detect C3. Exposure time for each blot was 1 s (**A**) and 10 min (**B**).

**Figure 6:**
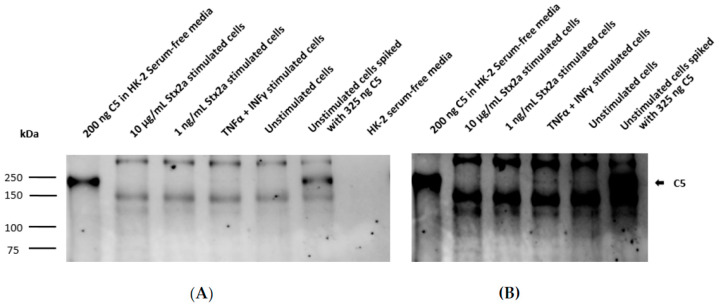
Secreted C5 by HK-2 cell line. Western blot results assessing extracellular protein secretion of C5 in HK-2 cells after stimulation for 9 h with 1 ng/mL and 10 µg/mL Stx2a. The presence of C5 was also studied in concentrated supernatants of TNF-α-and IFN-γ-stimulated cells and unstimulated cells (medium). Unstimulated cells spiked with 325 ng C5 or 200 ng of pure C5 were used as positive controls, while media was used as a negative control. Rabbit anti-human C5 antibody (Ab) was used to detect C5. Exposure time for each blot was 3 min (**A**) and 10 min (**B**).

Taking into account the results obtained from the ELISAs, intracellular C3 and secreted C5 were also studied by Western blot. Concentrated lysates or supernatants from unstimulated cells, TNF-α- and IFN-γ-stimulated cells and cells stimulated with the higher and lower Stx2a concentrations (10 µg/mL and 1 ng/mL, respectively) were further examined for the presence of C3 and C5. A total of 200 ng of pure C3b or C5 was used as reference. Western blot results for complement C3 protein synthesis were in line with the results obtained from the ELISA. C3 was only detected in the concentrated lysates of TNF-α- and IFN-γ-stimulated cells (Figure 5A) and in lesser amounts (at longer exposure time of the blot) in the unstimulated cells (Figure 5B). However, C3 could not be visualized in the lysates from cells stimulated with 1 ng/mL and 10 µg/mL of Stx2a (Figure 5). ELISA results from C5 secretion were also translated on a Western blot, in which a corresponding C5 band can just be suspected in the concentrated supernatants of TNF-α- and IFN-γ-stimulated cells and in the unstimulated cells, while no C5 bands were detected in the Stx2a-stimulated samples (Figure 6).

### 2.4. Stx2a Impact on the Viability of HK-2 Cells In Vitro

**Figure 7:**
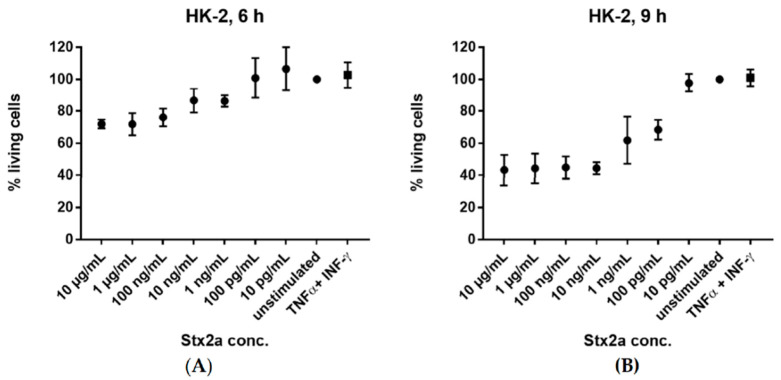
Cytotoxic activity of Stx2a. Percent of living HK-2 cells after 6 h (**A**) and 9 h (**B**) incubation with varying concentrations (10 pg/mL–10 µg/mL) of Stx2a are shown. Effect of TNF-α and IFN-γ stimulation on cell viability is also displayed. Viability of cells was measured by crystal violet staining after formalin fixation. Cells incubated with medium, in the absence of Stx2a or cytokines, were used as reference for a 100% viability. A sample size of *n* = 3–6 ± SD is shown.

We were curious whether Stx2a treatment had an impact on the viability of our cells that would simply preclude any protein synthesis. Thus, we examined the viability of HK-2 cells in vitro after stimulation with increasing concentrations of Stx2a, ranging from 10 pg/mL to 10 µg/mL. We also checked the effect of TNF-α and IFN-γ on cell viability. The cells were stimulated for 6 and 9 h. Cells with media, in the absence of Stx2a or cytokines, were used as reference for a 100% viability. HK-2 cells exhibited a gradual decrease in cell viability after 6 h stimulation with increasing Stx2a concentrations and showed a higher sensitivity to Stx2a after 9 h (Figure 7). In contrast, treatment with TNF-α and IFN-γ did not affect their viability even after 9 h.

## 3. Discussion

EHEC-associated HUS is an important cause of acute kidney injury, causing significant morbidity and mortality, particularly in childhood. Stx2a has been demonstrated to directly activate complement and to delay cofactor activity of fH in Chinese hamster ovary cells upon binding [17]. Further studies on the role of complement in eHUS revealed that Stx2a reduces expression of complement inhibitor CD59 on human renal tubular and glomerular endothelial cells [13]. The involvement of complement was corroborated by studies from Thurman and colleagues, who reported elevated levels of complement components Bb and soluble MAC (sC5b-9) in the serum of 17 eHUS patients [33]. Furthermore, alternative pathway activation by Shiga toxin was shown to trigger microvascular thrombosis [34], and there is evidence of platelet–leukocyte complexes forming with surface-bound complement components in the blood of eHUS patients [35].

We have demonstrated here that Stx2a binds to the two most central molecules of the complement cascade, C3b and C5. The role of this specific binding in the regulation of the complement cascade needs to be addressed in further studies. However, it seems more likely that the binding promotes complement activation, as previously shown [17], rather than its downregulation as shown for Staphylococcal complement inhibitors (SCIN) when binding to C3b and C3c [36]. Furthermore, the binding of the Stx2a to C3b and C5 could be involved in assisting the transport of the toxin through the body [37], or entrance into cells.

Immunoglobulins and complement component depositions are frequently found in glomeruli of diseased kidneys [38,39]. Moreover, the presence or absence of complement proteins in kidneys has been associated with acute renal transplant rejection [40,41] and with the development of renal injury [42,43]. However, the question remains whether these complement proteins are synthesized hepatically and thus acquired from the circulation, or are produced locally by intrinsic renal cells to compensate relative lack of plasma complement penetrating into tissues.

Therefore, we investigated whether extrahepatic cell lines, susceptible to Stx2, can induce local complement protein production under Stx2a stimulation. Indeed, increasing concentrations of Stx2a upregulated the expression of C3 and C5 mRNA in the tested cell lines. In HK-2 cells, Stx2a concentrations as low as 1 ng/mL were able to upregulate C3 and C5 expression. C3 and C5 mRNA expression was also upregulated in HCT-8 cells, but only upon stimulation with Stx2a concentrations greater than or equal to 1 µg/mL.

Other studies were also able to prove mRNA expression for complement genes, such as C2, C3 and C4 in kidney cells from nephritic kidneys [42], or even in kidney cells without any apparent stimulus [44]. These studies just focused on the transcriptome of the kidney cells. Nonetheless, to ascertain local complement synthesis, it is important to prove protein production, as shown by Andrew and co-workers for tubular epithelial and glomerular cells [45]. In our study, the observed upregulation of C3 and C5 mRNA expression in the HK-2 cells after stimulating with increasing concentrations of Stx2a for 6 h was not reflected at the protein level studied after 9 h of stimulation. Since increment of C3 protein level was observed in HK-2 cells stimulated with TNF-α and IFN-γ, we can assure the ability of this cell line to synthetize C3 and to regulate this production depending on certain stimuli. Detecting C5 in the supernatant of the HK-2 cells by ELISA while absent in the media (neg. control) itself suggests that HK-2 cells could also have the ability to synthetize C5, however, since none of the applied stimuli in this study was able to induce C5 protein synthesis, further studies would be necessary to ascertain this ability.

Our study showed a viability of 40–60% for HK-2 cells after 9 h stimulation with Stx2a concentrations of 1 ng/mL–10 µg/mL. This reduced, but still respective amount of viable cells present after 9 h excludes the notion that a lack of viable cells is the reason for the low and actually in our hands undetectable C3. Stx2a is known to cause ribosomal injury and block protein synthesis, which could explain why the significant increment of C3 and C5 mRNA levels in Stx2a-stimulated cells is not translated into an increase in protein levels. Alternatively, Satyam and colleagues showed that despite upregulation of C3 mRNA in intestinal epithelial Caco-2 cells during the ischemic phase of ischemia–reperfusion injury, C3 production was masked by intracellular cleavage of C3 by cathepsins [46]. The study reported that addition of cathepsin inhibitors resulted in reduction of C3a, suggesting that the cathepsins could be acting as complement convertases. The activation of complement proteins in the cytoplasm prior to their secretion could represent a possible new path of complement activation in contrast to the well-established canonical pathways of complement [18,46]. However, we have not addressed the formation of an intracellular alternative pathway C3 convertase; this could be subject to future investigations.

In conclusion, we could show a novel interaction between Stx2a and two central components of the complement cascade, C3b and C5. Furthermore, we observed upregulation of C3 and C5 mRNA in HK-2 and HCT-8 cells, which was, however, not evident at the protein level. The question of whether the observed interaction of C3b and C5 with Stx2a on protein levels leads to the previously reported activation of the complement system in the fluid phase in vitro has to be investigated in further studies. Moreover, the consequence of these protein interactions and of mRNA upregulation in the context of eHUS pathogenesis has to be addressed in advanced cell culture models or suitable animal models.

## 4. Materials and Methods

### 4.1. Reagents

Stx2a was purified as described by Brigotti et al. [47] referring to the Stx2a(cl), however, the *E. coli* strain C600 (933W) was used instead of *E. coli* R82 (pJES) 120DH5α. Briefly, the following steps were carried out: culture as described elsewhere [48], sonication, concentration with Vivaspin^®^ 6 columns (Sartorius, Goettingen, Germany) with an exclusion pore of 30 kDa, dialysis, hydroxyapatite chromatography, dialysis and concentration, followed by chromatofocusing using a diethylaminoethyl (DEAE) Sepharose fast flow ion exchanger [49], ammonium sulfate precipitation and dialysis. To remove endotoxin contaminants, an ActiCleanEtox column from Sterogene Bioseparations (Carlsbad, CA, USA) was used. The endotoxin concentration was then determined with an Endochrome-K^TM^ assay from Charles River (Dublin, Ireland), and found to be 23.16 EU/mL. The purity level of the purified protein was evaluated by SDS-PAGE. C3b and C5 were purchased from Sigma-Aldrich (St. Louis, MO, USA). Antibodies (Ab): rabbit anti-human C3c Ab and rabbit anti-human C5 Ab were from Agilent Dako (Santa Clara, CA, USA); mouse anti-human C3d Ab and mouse anti-human C3 Ab were from Santa Cruz Biotechnology (Dallas, TX, USA); sheep anti-human C5 Ab was from Abcam (Cambridge, UK); alkaline phosphatase-conjugated anti-rabbit Ig Ab and 4-nitrophenyl phosphate disodium salt hexahydrate (PNPP) were both from Sigma-Aldrich; horseradish peroxidase (HRP)-conjugated rabbit anti-mouse IgG and HRP-conjugated swine anti-rabbit IgG were both from Agilent Dako. TNF-α, IL-1β, IL-6, IFN-γ were purchased from Miltenyi Biotec (Bergisch Gladbach, Germany); PGE-2 was purchased from Sigma-Aldrich.

### 4.2. Cell Culture

Human colon epithelial HCT-8 cells were obtained from American Type Culture Collection—ATCC (ATCC^®^ CCL-244™) (Manassas, VA, USA) [50], and were maintained in RPMI-1640 medium (Sigma Aldrich) supplemented with fetal bovine serum to a final concentration of 10% at 37 °C under 5% CO_2_ conditions. Human proximal tubular epithelial HK-2 cells were obtained from Dr. Michael Joannidis (Medical University Innsbruck, Austria); the cells were cultured in supplemented growth medium consisting of a mixture of equal parts of DMEM and Ham’s F-12 nutrient mix (Thermo Fischer Scientific, Waltham, MA, USA) as described previously [51].

### 4.3. Analysis of Binding of Stx2a to Complement C3b and C5

Microtiter plates (96-well-medium binding microtiter plate, Greiner Bio-One, Kremsmünster, Austria) were coated with Stx2a (2 µg/well) in 100 µL coating buffer (0.15M NaHCO_3,_ 0.05M Na_2_CO_3_) overnight at 4 °C. After blocking with 1% (*w/v*) gelatin, each well was incubated with 0.5, 1 or 2 µg of C3b or C5 in 100 µL of TBST for 2 h at room temperature. After additional washing steps, bound C3b or C5 was incubated for 1 h at room temperature with a primary rabbit anti-human C3c Ab (1:500), which detects C3b or rabbit anti- human C5 Ab (1:200), followed by 1 h of incubation with alkaline phosphatase-conjugated goat anti-rabbit IgG Ab (1:1000). All antibodies were diluted in TBST. Detection was conducted with PNPP at OD 415/490 nm. Coated C3b or C5 (1 µg/well) were used as positive controls and BSA (2 µg/well) as negative control. To exclude cross reactivity between the antibodies and Stx2a, TBST instead of C3b or C5 was applied to Stx2a coated wells.

### 4.4. Alternative Pathway Intracellular Complement Profile Analysis of HK-2 Cells

HK-2 cells were grown to 80% confluence in 6-well plates (Costar Technologies, Coppell, TX, USA) and stimulated with different Stx2a concentrations from 1 to 100 ng/mL in fresh medium or medium in the absence of Stx2a for 6 h. Cells stimulated with TNF-α (5 ng/mL) and IFN-γ (10 ng/mL) were used as positive control for triggering C3, fB and fH production in human proximal tubular cells [28,29]. Cellular supernatants were collected and stored at −80 °C until analysis. Cells were trypsinized, and mRNA was extracted using the MagAttract Direct mRNA M48Kit (Qiagen, Hilden, Germany) and processed in the Thermo Scientific™ KingFisher™ mL Magnetic Purification System following the manufacturers’ instructions. The mRNA was quantified using Qubit™ RNA HS Assay Kit in a Qubit^®^ 2.0 Fluorometer (Thermo Fischer). The mRNA was normalized and reverse transcribed with the ABI 2700 PCR machine (Thermo Fischer) in 20 µL reaction volume. Gene expression was assessed by qPCR with TaqMan probes specific for *TOP2B*, *GAPDH*, *C3*, *C5*, *fH*, *fI*, *fB* and *fP* obtained from Thermo Fisher (Table 1). qPCR was performed in triplicate loading 1 ng of cDNA per reaction of volume of 10 µL and carried out on a QuantStudio™ 6 Flex Real-Time PCR System (Thermo Fisher). Reaction conditions included incubation at 50 °C for 2 min and 95 °C for 10 min followed by 40 cycles at 95 °C for 15 s and 60 °C for 1 min. PCR runs included no-template controls and no-reverse transcriptase controls. The target genes were normalized to housekeeping genes, *TOP2B* and *GAPDH*, and the results are expressed as fold changes from untreated cells using the ΔΔCt method.

### 4.5. Intracellular C3 and C5 mRNA Expression in HCT-8 Cells

HCT-8 cells were grown to 80% confluence in 6-well plates (Costar Technologies) and stimulated with different Stx2a concentrations from 0.001 to 10 µg/mL in fresh medium and medium in absence of Stx2a for 6 h. Cells stimulated with a cytokine cocktail of IL-1β (10^3^ U/mL), TNF-α (10 ng/mL), IL-6 (10^3^ U/mL) and PGE-2 (1 µg/mL) (ITIP) were used as a positive control for inducing complement C3 expression in human intestinal epithelial cells [31]. RNA was extracted here using the Total RNA Kit, peqGOLD (VWR, Radnor, PA, USA) following the manufacturer’s instructions. The mRNA was quantified using NanoVue™ Plus Spectrophotometer (GE Healthcare, Chicago, IL, USA). The mRNA was normalized and reverse transcribed using the iScript™ Reverse Transcription Supermix for qPCR following the manufacturer’s instructions. The mRNA expression of C3 was analyzed by qPCR using gene-specific primer/probe pairs. A GAPDH PCR using specific primer/probe pairs served as internal control to quantify the relative gene expression of the target genes. The SsoFast™ EvaGreen^®^ Supermix (Bio-Rad, Hercules, CA, USA) was used for target amplification, and runs were performed on the CFX96 real-time detection system (Bio-Rad). Reaction conditions included incubation at 50 °C for 5 min and 95 °C for 1 min followed by 40 cycles at 95 °C for 5 s and 60 °C for 5 s. The results were analyzed by the ΔΔCt method using the gene expression software of the cycler (CFX Manager Software version 2.1 by Bio-Rad).

### 4.6. Determination of C3 and C5 in HK-2 Cell Supernatants and Lysates Treated with Stx2a

HK-2 cells were grown to 80% confluence in tissue culture flasks 25 (300 cm^2^) (Techno Plastic Products AG, Trasadingen, Switzerland) and stimulated with Stx2a, medium in absence of Stx2a, or TNF-α (5 ng/mL) and IFN-γ (10 ng/mL) (see Section 4.4) for 9 h. Supernatants were recovered, centrifuged at 1500 g 10 min 4 °C, and stored at −20 °C until analysis. After washing with PBS, cells were lysed for 17 min with 1X mammalian cell lysis buffer (5X mammalian cell lysis buffer from Abcam), and the lysates were recovered. The lysates were then centrifuged at 1500 g for 5 min 4 °C, and the supernatants were stored at −20 °C until analysis (= lysate samples). For the positive control, lysates and supernatants of the cells incubated with medium in absence of Stx2a were spiked with 325 ng of C3b or C5 (Sigma-Aldrich) before storing at −20 °C until analysis. Supernatant samples were concentrated 10 times with Vivaspin^®^ 6 columns (Sartorius) with an exclusion pore of 30 kDa. Cell lysates and concentrated supernatants were tested for C3b and C5 with sandwich ELISA. ELISA: mouse anti-human C3d antibodies (1 µg/well) or sheep anti-human C5 antibodies (1 µg/well) in 100 µL coating buffer (see Section 4.3) were immobilized at 4 °C overnight in a high-binding microtiter plate (Greiner Bio-One). Incubations were performed at room temperature, if not indicated otherwise. After blocking with 1% (*w/v*) gelatin for 1 h, 100 µL of the sample was added and incubated for 2 h. Additionally, samples from unstimulated cells were spiked with commercial human C3b or C5 (Sigma-Aldrich) and used as positive controls, while mammalian cell lysis buffer and cell medium buffer instead of the sample were used as negative controls. Bound C3 or C5 were detected with rabbit anti-human C3 Ab (also detecting C3b; 1:500) or rabbit anti-human C5 Ab (1:200), followed by an alkaline phosphatase-conjugated goat anti-rabbit IgG Ab (1:1000), all diluted in TBST. Detection was performed with PNPP at OD 415/490 nm. The cut-off value of the ELISA was calculated by the mean of the negative control + 2.6 times its SD [32]. Concentrated lysates or supernatants from unstimulated cells, TNF-α-and IFN-γ-stimulated cells and cells stimulated with the higher and lower Stx2a concentrations (10 µg/mL and 1 ng/mL, respectively) were further examined for the presence of C3 and C5 by Western blot. Western blot: lysates or concentrated supernatants were further concentrated with Vivaspin^®^ 500 columns (Sartorius) with exclusion pore of 10 kDa. The concentrated samples were then electrophoresed through 10% SDS-bis-acrylamide gels (Sigma Aldrich), under non-reducing conditions. Proteins were transferred to a polyvinylidene fluoride (PVDF) membrane (Bio-Rad) and, after blocking, incubated with mouse anti-human C3 Ab or rabbit anti-human C5 Ab at 4 °C overnight. Bound Abs were detected with rabbit anti-mouse IgG or swine anti-rabbit IgG (both HRP-conjugated) for 1 h, followed by chemiluminescent visualization with enhanced chemiluminescence (ECL) substrate (Bio-Rad) in the ImageQuant Las 4000. Spiked samples with 325 ng C3b or C5 were used as positive control, 200 ng of pure C3b and C5 (Sigma-Aldrich) were loaded onto the gel as reference, and mammalian cell lysis buffer and cell medium buffer instead of the sample were used as negative controls.

### 4.7. Cytotoxicity Assay in HK-2 Cells

The cytotoxicity assay was performed to determine the toxic effect of Stx2a on the used cell lines, as described previously [4]. TNF-α and IFN-γ effect on HK-2 cells viability was also analyzed. HK-2 cells were seeded in 96-well plates (Greiner Bio-One) by up to 80% confluency and incubated at 37 °C and 5% CO_2_ until they reached 100% confluency. The cells were then stimulated with Stx2a concentrations of 10 pg/mL–10 µg/mL in fresh medium, TNF-α (5 ng/mL) and IFN-γ (10 ng/mL) in fresh medium, and medium with absence of Stx2a or cytokines for 6 and 9 h. After washing with PBS, the cells were fixed with 70 µL of 2% formalin for 3 min and stained with 70 µL of 1% crystal violet for 1 h. After washing with distilled water, 100 µL of 50% ethanol was added to the wells for 45 min at shaking conditions. The absorbance was measured at 550 nm as an estimate of the density of cells that remained in the wells. Cells incubated with medium only—without the presence of Stx2a or cytokines—were used as reference and set to a 100% viability.

### 4.8. Statistical Analyses

The results were analyzed with GraphPad Prism (version 7) software. Unpaired Student’s *t*-test corrected with Bonferroni–Holm correction for multiple comparisons was used for analyzing the transcriptome data. One-way ANOVA corrected with Tukey correction for multiple comparison was performed to compare the read-out parameters of the binding experiments, as well as the protein expression. The level of significance was set to α = 0.05.

## Figures and Tables

**Table 1:** Gene name, gene symbol and assay ID from TaqMan to assess gene expression by real-time PCR.

Gene Name	Gene Symbol	Assay ID
Topoisomerase (DNA) II beta	*TOP2B*	Hs00172259_m1
Glyceraldehyde-3-phosphate dehydrogenase	*GAPDH*	Hs02786624_g1
Complement component 3	*C3*	Hs00163811_m1
Complement component 5	*C5*	Hs01004342_m1
Complement factor H	*CFH*	Hs00962373_m1
Complement factor I	*CFI*	Hs 00989715_m1
Complement factor B	*CFB*	Hs00156060_m1
Complement factor P	*CFP*	Hs01106971_g1

## Data Availability

Data are available upon request, please contact the contributing authors.
